# Social structure and *Escherichia coli* sharing in a group-living wild primate, Verreaux’s sifaka

**DOI:** 10.1186/s12898-016-0059-y

**Published:** 2016-02-12

**Authors:** Andrea Springer, Alexander Mellmann, Claudia Fichtel, Peter M. Kappeler

**Affiliations:** Behavioral Ecology and Sociobiology Unit, German Primate Center, Kellnerweg 4, 37077 Göttingen, Germany; Institute of Hygiene, University Hospital of Münster, Robert-Koch-Straße 41, 48149 Münster, Germany; Department of Sociobiology and Anthropology, University of Göttingen, Kellnerweg 6, 30077 Göttingen, Germany

**Keywords:** Fecal-oral disease transmission, *Propithecus verreauxi*, *Escherichia coli*, Multilocus sequence typing, Scent-marking, Social contacts, Social network analysis, Spillover

## Abstract

**Background:**

Epidemiological models often use information on host social contacts to predict the potential impact of infectious diseases on host populations and the efficiency of control measures. It can be difficult, however, to determine whether social contacts are actually meaningful predictors of transmission. We investigated the role of host social structure in the transmission of *Escherichia coli* in a wild population of primates, Verreaux’s sifakas (*Propithecus verreauxi*). Using multilocus sequence typing (MLST), we compared genetic similarities between *E. coli* isolates from different individuals and groups to infer transmission pathways.

**Results:**

Correlation of social and transmission networks revealed that membership to the same group significantly predicted sharing of *E. coli* MLST sequence types (ST). Intergroup encounter rate and a measure of space-use sharing provided equally potent explanations for type sharing between social groups when closely related STs were taken into account, whereas animal age, sex and dispersal history had no influence. No antibiotic resistance was found, suggesting low rates of *E. coli* spillover from humans into this arboreal species.

**Conclusions:**

We show that patterns of *E. coli* transmission reflect the social structure of this group-living lemur species. We discuss our results in the light of the species’ ecology and propose scent-marking, a type of social contact not considered in previous epidemiological studies, as a likely route of transmission between groups. However, further studies are needed to explicitly test this hypothesis and to further elucidate the relative roles of direct contact and environmental transmission in pathogen transfer.

**Electronic supplementary material:**

The online version of this article (doi:10.1186/s12898-016-0059-y) contains supplementary material, which is available to authorized users.

## Background

Pathogens and parasites—broadly defined as organisms that live on and draw nutrients from a host [1]—may impact their hosts’ survival and fitness considerably [1–3], often leading to declines in wildlife populations [e.g. 4–6]. Epidemiological models predict the potential impact of infectious diseases on host populations and the efficiency of control measures. However, whereas most traditional models have assumed homogeneous mixing of individuals within a host population [7], it is now recognized that heterogeneity in contact patterns arising from both the social behavior of individual hosts and the spatial structure of host populations affect pathogen transmission [8–11]. Epidemiological models accounting for social contact heterogeneities in wildlife have been introduced in the 2000s [e.g. 12–14] and may yield predictions that differ dramatically from mean-field models assuming homogeneous mixing [15, 16], especially with regard to threshold population sizes for disease invasion [17] as well as transmission [18] and mortality rates [19].

Therefore, increasing efforts are being made to accurately assess transmission-relevant social contact patterns in humans [20] and wildlife [21] through direct observations [22], live-trapping [23] or the use of proximity loggers [20, 24]. It can be difficult, however, to establish whether the contact patterns that have been measured are meaningful for actual transmission [20]. Moreover, it remains a major goal in disease ecology to determine the relative importance of social pathogen transmission compared to uptake from environmental reservoirs [25–27].

*Escherichia coli* is an ideal model organism to trace transmission of fecal-orally transmitted microorganisms [28]. It is the main facultative anaerobic commensal colonizing the gastrointestinal tract of mammals and exhibits a clonal population structure that is little affected by genetic transfer and mutation [29]. Healthy humans and dogs usually carry one predominant, resident strain of *E. coli*, which is present for months to years, and one to several transient strains [30–32]. Thus, if two individuals share the same or genetically very similar strains, it can be assumed that either direct transmission has occurred between them, or that they have been exposed to a common source of *E. coli* [28].

Among wild mammals, concordance between contact networks and *E. coli* strain sharing networks has been demonstrated in the fission–fusion society of giraffes [26], in a multihost system of African ungulates [33] and in solitary mountain brushtail possums [34]. A similar study in elephants has been hampered by the fact that *E. coli* survive in water sources, which can act as infection reservoirs, masking social transmission [25]. None of these studies however, examined the relationship between social networks and bacterial transmission across multiple discrete, adjacent social groups, which is a common setting in epidemiological models [35–38].

We studied social determinants of *E. coli* transmission in 10 groups of Verreaux’s sifakas (*Propithecus verreauxi*), an endemic Malagasy primate. Verreaux’s sifakas are diurnal, folivorous lemurs that are strictly arboreal. They live in small stable, multi-male multi-female groups and defend core areas of their home range by scent-marking in overlap areas of regular intergroup encounters [39, 40]. Males disperse from their natal group at around 3–5 years of age [41]. In contrast to most other primates, the small group and home range size of sifakas permit simultaneous study of multiple neighboring groups [e.g. 39, 40], and thus a rare opportunity to study transmission within and between social groups of primates in a natural setting.

Microorganisms can be taken up directly from the environment or through contact with members of other species, potentially masking social transmission between conspecifics. However, because Verreaux’s sifakas do not drink from waterholes, but rely on licking dew from trees as well as on the water content of their diet, *E. coli* transmission via environmental reservoirs is unlikely. Furthermore, environmental conditions in the habitat are considered unfavorable for *E. coli* survival during most of the year [42, 43], as a long dry season is accompanied by high-amplitude daily temperature fluctuations, high levels of light exposure and low humidity [41].

We investigated whether *E. coli* transmission, measured as multilocus sequence type (MLST ST) sharing, is influenced by group membership, GPS-derived intergroup encounter rates and a measure of space-use sharing between groups, the Utilization Distribution Overlap Index (UDOI), while controlling for host sex, age and dispersal history. If direct social contacts were important for *E. coli* transmission, we predicted that ST sharing would be more prevalent within than between groups, and we expected intergroup encounter rates to better explain strain-sharing than joint space-use.

While *E. coli* transmission from environmental reservoirs may be unlikely, contact with humans represents a potential source of pathogens, as the study population has been exposed to visits by several thousand tourists per year. It has been shown that other lemurs living in habitats frequented by tourists harbor pathogenic *Enterobacteria,* which do not occur in undisturbed populations [44]. *E. coli* serves as an important indicator of potential microbial spillover from humans or livestock into wildlife populations [45, 46]. Although *E. coli* is most often a commensal, many pathogenic strains exist, causing severe intestinal and extra-intestinal disease [47, 48], also in lemurs [49]. Among these, multi-resistant, extended-spectrum beta-lactamase (ESBL)-producing strains have recently emerged worldwide as important causes of disease both in humans and animals [50, 51]. By testing for antibiotic resistance of *E. coli* isolates, we assess the risk of microbial spillover from humans to a species belonging to the most endangered group of mammals, the lemurs of Madagascar [52].

## Methods

### Study area and host population

The study was carried out in Kirindy Forest, Western Madagascar, located at 44°39′E, 20°03′S. The 90-ha study area is part of a field site operated by the German Primate Center, where lemurs are habituated and individually marked with unique collars, including radio (Holohil Systems, Carp, Ontario, Canada) and GPS (e-obs, Grünwald, Germany) units, as part of an ongoing long-term study [41], which has been approved by the Ethics Committee of the German Primate Center and the Ministère des Eaux et Forêts of Madagascar. All necessary research permits were obtained from the respective Malagasy and German authorities (Ministère des Eaux et Forêts of Madagascar; Commission ad hoc Flore et Faune (CAFF) of Madagascar; Centre National de Formation, d’Etudes et de Recherche en Environnement et Foresterie (CNFEREF); The Federal Agency for Nature Conservation of Germany). Regarding animal welfare, we followed the “Code of Best Practices for Field Primatology 2014” of the International Primatological Society.

We studied 10 social groups of Verreaux’s sifakas, 8 of which were adjacent. Samples from two groups living about 2 km away from the principal study population were also included. Groups ranged in size from 3 to 7 individuals, comprising 36–38 animals in total. Censuses of group membership are carried out two to three times a week [41].

### Estimation of home range size, home range overlap and intergroup encounter rates

One adult animal in each of the 8 adjacent groups was equipped with a GPS collar. Scan sampling of group cohesion during animal observations showed that all other group members were in a radius of less than 10 m from collared individuals in more than 50 % of the time (range 53–90 %, mean 69.4 %), thus we considered ranging data from those individuals to be representative for the group as a whole. Animals considered likely to roam (i.e. young males) were not chosen to be collared. Collars were set to simultaneously record GPS coordinates every 15 min, from 04:00 to 20:00 h local time. GPS data were collected from August to December 2013 and from March to July 2014. GPS data were not available for the two groups living outside of the principal study area.

We calculated 95 % kernel home ranges and their overlaps for bi-weekly intervals using the adehabitatHR package [53] in R version 3.0.2. To quantify space-use sharing between the different groups, i.e. how much the animals actually use the overlap area, we calculated the Utilization Distribution Overlap Index (UDOI) [54]. This index usually ranges from 0 to 1 but can be >1 if overlap is high and space-use is non-uniformly distributed. To derive intergroup encounter rates from the GPS data, we used the linear movement model contained in the R package movement Analysis [55], assuming linear movement between location measurements. An encounter was inferred if two groups were in ≤42 m distance based on the interpolated trajectories. The 42 m distance threshold was derived by calculating the distance between the groups’ GPS locations during directly observed intergroup encounters, based on an extended dataset of observations and GPS data collection over the course of one year [56]. Using this threshold best predicted observed encounters. A new encounter was recorded if the two GPS-bearing individuals from different groups were at a distance >42 m for at least 30 min, until this threshold was crossed again.

Encounter rates were calculated as encounters per day for bi-weekly intervals. We tested for correlation between UDOI and intergroup encounter rate using Spearman rank correlation.

### Direct observations

In order to collect data on animal behavior during encounters, direct observations were conducted in the 8 adjacent groups during two periods, once during the dry, lean season from August to October 2013 and once during the wet season from February to May 2014. Focal animal observations of 1 h per individual were carried out in an alternating order for 3 h in the morning and 3 h in the afternoon, resulting in 4 statistical days per individual, which amounted to a total of 860 h of focal animal observations. During intergroup encounters, the identities and proportion of participating animals, all close contacts (i.e. grooming, body contact or proximity of <1 m) between members of different groups and their durations as well as the total duration of the encounter were recorded ad libitum.

### Sample collection, *E. coli* isolation and genotyping

Rectal swabs (Transwab^®^ Amies, Medical Wire and Equipment, Corsham, Wiltshire, UK) were taken in the course of routine biomedical examinations of immobilized animals. Fecal samples were collected within 2 min of defecation from those individuals from whom rectal swabs could not be obtained. Samples were obtained during 3 periods, each spanning at most 25 days: In March and April 2013 and in August 2013, we took rectal swabs from 14 and 11 individuals, respectively, and in March and April 2014, 24 rectal swabs and 15 fecal samples were taken, resulting in a total of 66 samples from 48 individuals. Individuals from all groups were sampled in random order in each period (Additional file 1), to exclude the possibility that MLST sharing within groups could arise as an artifact of the time point of sampling. Four individuals were repeatedly sampled. Pre-cultivation of bacteria was undertaken in the field laboratory to ensure that a sufficient number would survive storage and transport and thus maximize *E. coli* recovery: Rectal swabs were streaked within 48 h onto MacConkey and Columbia blood agar and used to inoculate glucose-containing nutrient broth (agar and broth: Oxoid GmbH, Wesel, Germany). Broth and agar plates were incubated for 24–32 h at 37 °C. After this first incubation period, broth was streaked onto both MacConkey and Columbia blood agar and incubated for another 24 h. To maximize recovery of *E. coli*, we randomly picked several colonies from all four agar plates, representing all colony morphology types present on the plate, dissolved them in sterile 0.9 % sodium chloride solution with an addition of 20 % glycerol and froze them at −20 °C until shipment and further processing. Fecal samples were treated slightly differently: They were used to inoculate glucose-containing nutrient broth, and after incubation of 24–32 h, an aliquot of the broth was frozen at −20 °C with an addition of 20 % glycerol.

In the laboratory in Germany, samples were streaked out onto MacConkey and Columbia blood agar. After an incubation period of 24–48 h, up to 4 colonies typical for *E. coli* were isolated per sample and subjected to species identification using matrix-assisted laser desorption ionization time-of-flight mass-spectrometry (MALDI-ToF MS; Bruker GmbH, Bremen, Germany). Antimicrobial susceptibility testing was done by agar disc diffusion as recommended by the European Committee on Antimicrobial Susceptibility Testing (EUCAST) applying EUCAST clinical breakpoints for categorization of susceptible, intermediate and resistant isolates. All isolates were tested for multidrug resistance due to the production of extended-spectrum beta-lactamases (ESBL) using a chromogenic agar plate (chromID™ESBL; Bio Mérieux, Marcy l’Etoile, France). Isolates belonging to ST131 were additionally tested for resistance against the following antibiotics: Ampicillin, Piperacillin, Cefuroxim, Cefotaxim, Cefpodoxim, Ceftazidim, Cefepime, Piperacin/Tazobactam, Imipenem, Meropenem, Ertapenem, Trimethoprim/Sulfamethoxazol, Tigecyclin, Gentamicin, Amikacin, Ciprofloxacin, Fosfomycin and Nitrofurantoin. These include, but are not limited to, the most prevalent resistance-causing antibiotics used in Madagascar [57].

For molecular subtyping, each isolate was characterized using MLST [58]. This typing method relies on determination of the sequence of internal fragments of seven housekeeping genes [59] and STs were assigned according to the *E. coli* MLST website (http://mlst.warwick.ac.uk/mlst/dbs/Ecoli). Sequences were further analyzed using the SeqSphere^+^ software version 1 (Ridom GmbH, Münster, Germany). The minimum spanning tree based on the MLST was generated also using the SeqSphere^+^ software.

### Construction of the transmission network

We constructed an *E. coli* transmission network by assigning a link to a dyad if the two animals shared the same ST. Links were unweighted, i.e. we did not make a distinction between the number of types that animals shared, and undirected as we did not make any assumptions about the direction of transmission. We also constructed a second network, taking strains that only differed in one of the seven loci (single locus variants, SLV) into account as evidence for less recent transmission. Animals from which we did not obtain any isolate were not included.

### Statistical analyses

We tested for significant differences in *E. coli* recovery rate between the two sampling methods, rectal swab and fecal sample, using a χ^2^-test. We then tested whether the number of STs isolated from an individual was significantly correlated with the animals’ age, the size of its’ social group and sampling effort, i.e. the number of samples that were taken from this individual, using Spearman rank correlations. Because adult males are expected to roam more than adult females, we tested for differences in the number of sequence types between adult males and adult females using a Wilcoxon rank sum test. We used nonparametric tests, because the data violated assumptions of generalized linear mixed models.

To determine which factors influence the likelihood of a link occurring in the transmission network, we analysed pair-wise strain-sharing using Bayesian regression modelling. Because of the non-independence of network data, statistical methods that assume data independence are not appropriate [60]. We controlled for non-independence by modelling the identities of animals as random effects with a multi-membership structure in generalized linear mixed models (logit link) using the R package MCMCglmm [61].

We investigated in a multivariate model whether the following factors

- belonging to the same sex,
- being born in the same year,
- being born in the same social group,
- being a member of the same social group at the point of sampling,
- having been sampled in the same month and
- having been sampled by the same method (rectal swab or fecal sample)

influenced a dyad’s log-odds of having the same *E. coli* ST.

Including only the 8 neighboring groups, we further tested whether UDOIs and intergroup encounter rates were significantly correlated with *E. coli* type sharing between dyads belonging to different groups. For UDOIs as well as encounter rates, we used the arithmetic mean of all bi-weekly measurements to best represent the relation between the two groups throughout the study period, and we z-transformed those values to a mean of zero and a standard deviation of one for better comparability. We constructed separate models for these 2 predictors because of their strong correlation, and controlled for birth cohort (i.e. same age), sex, natal group, sampling month and sampling method. In a second set of models, we included sharing of single locus variants as evidence for less recent transmission.

We used non-informative priors and fixed the observation-level variance (R-structure) at 1. We ran the models for 1,000,000 iterations, with a thinning parameter of 500 and a burn in phase of 10,000 to achieve low autocorrelation between recorded iterations. This resulted in a sample size of 1980 for estimating the posterior distribution. All statistical analyses were performed in R v. 3.2.2.

## Results

### Home range overlap and intergroup encounters

**Fig. 1:**
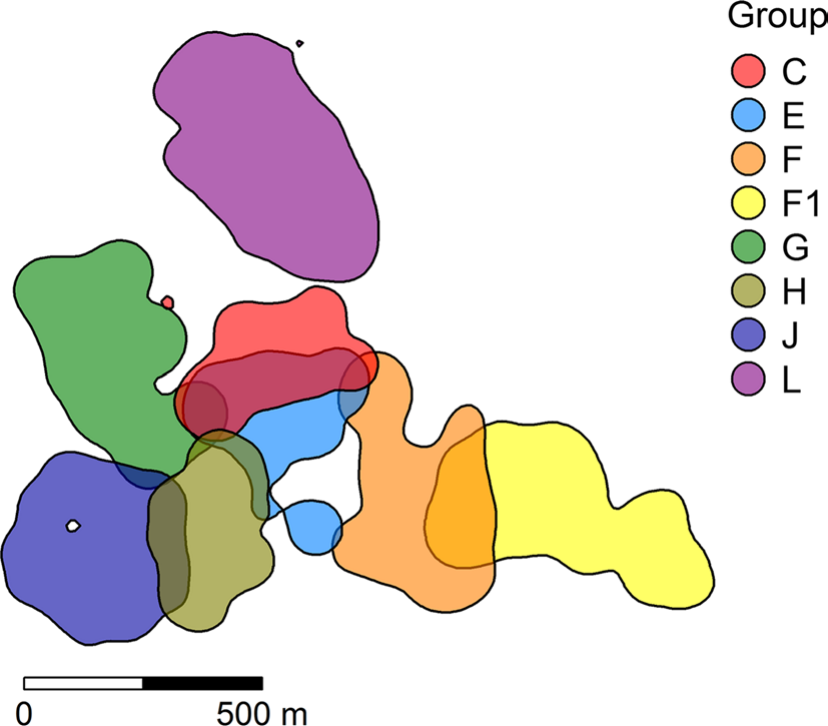
95 % kernel home ranges of the eight adjacent *Propithecus verreauxi* groups in Kirindy Forest, Madagascar. Home ranges were calculated for biweekly intervals. Here, home ranges from the first half of May 2014 are shown

Mean bi-weekly 95 % kernel home ranges varied in size from 5.67 to 14.15 ha among groups, and the corresponding pair-wise home range overlap of the 14 overlapping dyads varied between 0.64 and 3.88 ha (Additional file 2). Mean UDOIs of neighboring groups varied from 0.00017 to 0.098. Home ranges and overlaps from one exemplary bi-weekly period are shown in Fig. 1.

A total of 581 encounters were inferred based on GPS data, and mean bi-weekly intergroup encounter rates varied from 0.005 to 0.7 encounters per day. Fourty-nine intergroup encounters (8.4 % of all encounters) were directly observed. Mean duration (±SD) of observed intergroup encounters was 12.5 ± 11.5 min (range 2–60 min). Scent-marking occurred in 75.5 % of those encounters, while proximity <1 m between members of different groups occurred in 32.7 % and body contact and grooming only occurred during 16.3 and 12.2 % of encounters, respectively. Mean UDOIs and mean intergroup encounter rates of the 14 neighboring group-dyads were strongly correlated (Spearman rank correlation, N = 14, S = 12, rho = 0.97, P < 0.001).

### Recovery of *E. coli* types

**Fig. 2:**
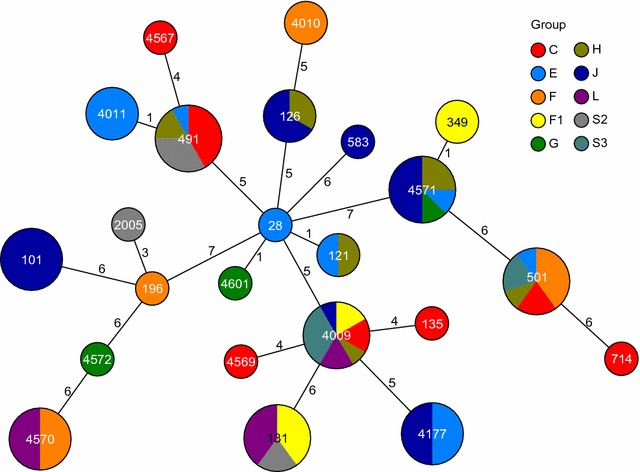
Minimum spanning tree based on the allelic profiles of the 24 MLST STs isolated. Node size is proportional to the frequency of sequence occurrence, numbers on connecting lines are the number of differing alleles in a pair-wise comparison, and *colors* indicate host social group

We characterized 83 *E. coli* isolates, belonging to 39 individuals from 10 social groups (mean 1.7 isolates per individual, range 0–6). Twenty-nine individuals were natal to their social group, while 10 animals (one female, nine males) were immigrants. Isolation success did not differ significantly between rectal swabs and fecal samples (76.5 % of N_rectal_ = 51 vs. 60 % of N_fecal_ = 15, χ^2^ = 0.78, P = 0.37). We isolated 24 distinct MLST STs, 13 of which (54.2 %) occurred in multiple hosts (Fig. 2). Previously unknown STs were deposited in the *E. coli* MLST database (http://mlst.warwick.ac.uk/mlst/dbs/Ecoli). Up to 2 different STs were obtained per sample and up to 4 different STs per individual host (mean: 1.25 STs). Of the 4 animals that were sampled repeatedly, we only once found the same type twice in subsequent samples. All isolates were non-ESBL-producing. Further susceptibility tests on isolates of type ST131 (N = 5), an ST that is shared with the major antibiotic resistant *E.* *coli* lineage [62], revealed full susceptibility to all antimicrobial agents tested.

### Predictors of individual ST richness and sharing

There was no significant correlation between the number of STs isolated from an individual and its age, the size of its social group, or the number of samples collected from an individual (Spearman rank correlation, rho = 0.05, P = 0.76; rho = −0.12, P = 0.42; rho = 0.25 and P = 0.08, respectively). Adult males did not harbor more STs than adult females (Wilcoxon rank sum test, W = 183.5, P = 0.54).

**Fig. 3:**
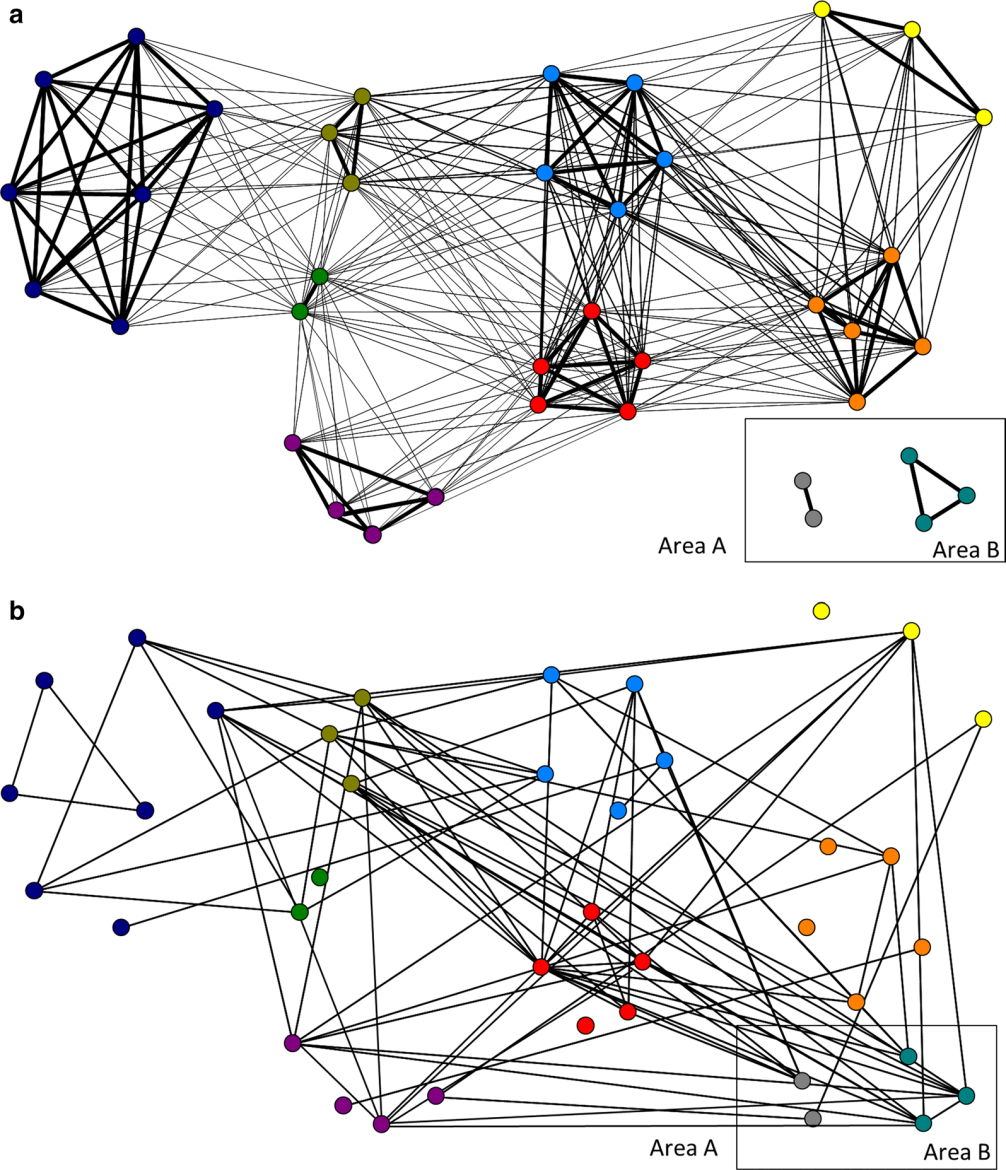
Networks including 10 social groups from 2 study areas based on **a** group membership and home range overlap and **b** *E. coli* ST sharing. Nodes are arranged by social groups, indicated by the *different colors*. In network **a** thickness of lines is proportional to the degree of home range overlap (within-group overlap = 100 %) while in network **b** lines indiscriminately indicate that two individuals share the same *E. coli* ST

**Fig. 4:**
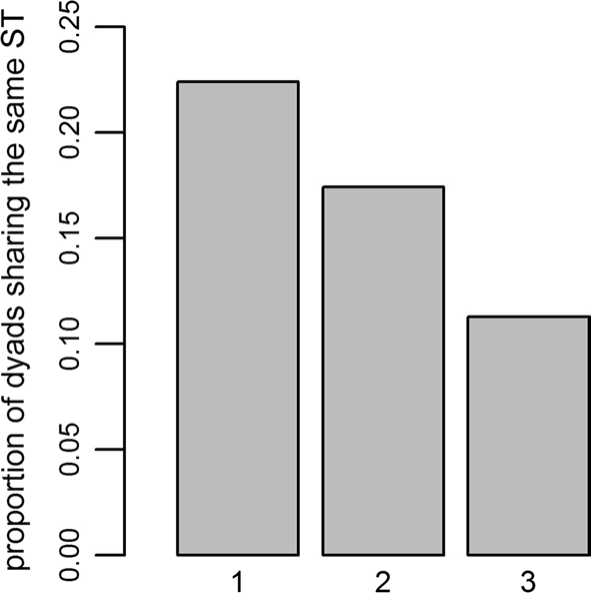
Proportion of dyads sharing the same *E. coli* sequence type in each of three association classes: *1* Belonging to the same group (N = 67), *2* belonging to adjacent groups whose ranges overlap (N = 224) and *3* belonging to non-adjacent groups (N = 443)

Density of the *E. coli* transmission network was 0.116 (Fig. 3b), indicating that 11.6 % of all possible connections were present. Dyads that belonged to the same social group were more likely to share the same or closely related STs as compared to dyads from different social groups with home range overlap (Fig. 4). We examined whether this pattern might be driven by mother-offspring ST-sharing, but none of the 10 mother-offspring dyads in the dataset shared the same ST. The likelihood of sharing STs was smallest for animals belonging to non-neighboring groups. We tested whether several dyad-level attributes influenced the likelihood of a link occurring between two individuals, but the full *E. coli* transmission network was only significantly correlated with the group membership network; all other predictor variables were not statistically significant (Table 1, model A). In fact, belonging to the same social group improved the odds of sharing the same ST 3.1 times. Regarding links between social groups, none of the variables tested were statistically significant predictors of sharing the same ST (Table 1, models B and C). However, in the second set of models which included sharing of closely related *E. coli* STs, UDOIs and intergroup encounter rates were strongly correlated with the intergroup transmission network (Table 2, models B and C), qualifying both as conduits of social transmission between different groups. Furthermore, the effect sizes of these parameters were almost identical. An increase of one standard deviation in home range overlap increased the odds of sharing the same or a closely related ST 1.54 times, whereas an increase of one standard deviation in intergroup encounter rates resulted in 1.58 times higher odds. Belonging to the same birth cohort was also significant, but the correlation coefficient was negative and the corresponding odds ratio <1 in both models, indicating that in this data subset, animals born in the same year had a smaller chance of harboring the same *E. coli* strain than by chance.

**Table 1:** Bayesian general linear mixed models testing the influence of several predictor variables on the probability of sharing the same *E. coli* MLST

Model	Predictor	Posterior mean coefficient	95 % Confidence interval	MCMCglmm *P* value	Odds ratio^a^
A	Intercept	−3.28	−4.4, −2.24	*<0.001*	
(DIC: 402.95)	Same sex	0.05	−0.61, 0.67	0.864	1.05
	Same birth year	−0.5	−2.05, 1.03	0.534	0.6
	Same natal group	0.06	−1.31, 1.22	0.915	1.06
	Same group	1.14	0.02, 2.24	*0.044*	3.13
	Same sampling month	0.21	−0.47, 0.94	0.552	1.23
	Same sampling method	0.03	−0.86, 0.94	0.926	1.03
B	Intercept	−3.75	−5.4, −2.21	*<0.001*	
(DIC: 243.32)	Same sex	−0.38	−1.18, 0.46	0.366	0.68
	Same birth year	−2	−4.99, 0.54	0.119	0.14
	Same natal group	1.12	−0.86, 3	0.235	3.06
	Space-use sharing (UDOI)	0.13	−0.32, 0.57	0.538	1.14
	Same sampling month	0.66	−0.31, 1.63	0.177	1.93
	Same sampling method	−0.2	−1.35, 0.93	0.727	0.82
C	Intercept	−3.74	−5.21, −2.27	*<0.001*	
(DIC: 243.17)	Same sex	−0.39	−1.23, 0.47	0.347	0.68
	Same birth year	−2.04	−4.87, 0.66	0.107	0.13
	Same natal group	0.95	−1.03, 2.85	0.329	2.59
	Intergroup encounter rate	0.2	−0.24, 0.64	0.346	1.22
	Same sampling month	0.64	−0.33, 1.57	0.190	1.9
	Same sampling method	−0.18	−1.29, 0.98	0.769	0.84

Models B and C included only the 8 neighboring groups and excluded ties within the same social group. UDOI and intergroup encounter rate were z-transformed. Significant P values (<0.05) are printed in *italics*

^a^The odds ratio is the multiplicative increase in the odds of *E. coli* strain sharing with each unit increase in the explanatory variable

**Table 2:** Bayesian general linear mixed models testing the influence of several predictor variables on the probability of sharing the same *E. coli* MLST or a single locus variant

Model	Predictor	Posterior mean coefficient	95 % Confidence interval	MCMCglmm P value	Odds ratio^a^
A	Intercept	−2.64	−3.63, −1.74	*<0.001*	
(DIC: 474.2)	Same sex	−0.27	−0.83, 0.26	0.346	0.76
	Same birth year	−0.46	−1.83, 0.81	0.502	0.63
	Same natal group	0.09	−1.07, 1.26	0.879	1.09
	Same group	1.04	0.1, 2.16	*0.048*	2.83
	Same sampling month	0.05	−0.59, 0.67	0.879	1.05
	Same sampling method	0.05	−0.78, 0.8	0.88	1.05
B	Intercept	−2.87	−4.12, −1.64	*<0.001*	
(DIC: 305.96)	Same sex	−0.34	−1.04, 0.35	0.354	0.71
	Same birth year	−2.36	−5.2, 0.15	*0.037*	0.09
	Same natal group	0.55	−1.24, 2.38	0.565	1.73
	Space-use sharing (UDOI)	0.43	0.07, 0.78	*0.021*	1.54
	Same sampling month	0.22	−0.65, 1.04	0.585	1.25
	Same sampling method	−0.12	−1.06, 0.9	0.831	0.89
C	Intercept	−2.91	−4.22, −1.71	*<0.001*	
(DIC: 305.87)	Same sex	−0.31	−1.05, 0.37	0.386	0.73
	Same birth year	−2.31	−5.15, 0.18	*0.046*	0.1
	Same natal group	0.32	−1.8, 2.16	0.741	1.38
	Intergroup encounter rate	0.46	0.12, 0.81	*0.015*	1.58
	Same sampling month	0.18	−0.7, 1	0.665	1.2
	Same sampling method	−0.05	−1, 1.02	0.949	0.95

Models B and C included only the 8 neighboring groups and excluded ties within the same social group. UDOI and intergroup encounter rate were z-transformed. Significant P values (<0.05) are printed in *italics*

^a^The odds ratio is the multiplicative increase in the odds of sharing the same or a closely related ST with each unit increase in the explanatory variable

## Discussion

Using *E. coli* as a model for other, potentially more harmful fecal-orally transmitted microorganisms, this study revealed that the social structure of a group-living primate shapes pathogen transmission. Group membership was the single best explanatory variable for *E. coli* strain sharing, while both intergroup encounter rates and space-use sharing qualified as predictors for inter-group transmission when single locus variants of *E. coli* STs were taken into account as evidence for less recent transmission. These measures of sociality should therefore be considered in epidemiological modeling of fecal-orally transmitted infectious agents. In contrast, there was no evidence for *E. coli* spillover from humans, despite exposure to anthropogenic activities, including human defecation, in the study area. The strictly arboreal lifestyle of sifakas, in combination with reliance on ephemeral water sources, may therefore hamper environmental uptake of *E. coli* and other similarly transmitted microorganisms, although comparison with a sympatric lemur species that spends more time on the ground (*Eulemur rufifrons*) will be needed to confirm this hypothesis [cf. 63].

Extensive *E. coli* strain sharing has been documented in members of the same household, including pets [64, 65], suggesting that households function as a microbiological unit. Our results show that wild groups mirror these microbiological units in Verreaux’s sifakas. Belonging to the same social group was the only significant predictor of *E. coli* ST sharing as compared to possible non-social influences such as host sex and age, which were important determinants of *E. coli* community composition in elephants [25]. Being born in the same group also had no significant effect, indicating that adult animals acquire the *E. coli* types of their new group after dispersal, and that their *E. coli* community is not static throughout life, but responds to changing social conditions, as in humans [66]. Belonging to the same birth cohort even exhibited a negative correlation with *E. coli* type sharing. This makes sense in light of the fact that in most years, one or two juveniles are born into each social group. Thus, each birth cohort consists of members of different groups, which have a higher likelihood of harboring the same *E. coli* type as their group members rather than that of conspecifics of the same age.

We recovered a maximum of 4 different STs from an individual and this number adequately represented within-host *E. coli* diversity found in humans [30] and other animals [26, 31]. One limitation of the study, however, is that we were unable to assess strain-turnover in individual hosts, as only 4 animals could be sampled more than once due to practical constraints of studying wild primates. In those animals, we only once recovered the same ST twice. Future research will have to reveal whether this was due to strain-turnover, variation in strain abundance within the host, or the fact that sometimes subdominant strains can be more easily cultured than the dominant, resident strain [67]. However, *E. coli* strains have been shown to persist for 1–3 years in humans and their pets [65].

Furthermore, if strain-turnover on the population level was indeed high, sampling during different periods should obscure any social signature rather than strengthen it, especially since sampling was equally distributed over all of the groups during each sampling period. To investigate whether our results were influenced by the different sampling periods or by the fact that we used both rectal swabs and fecal samples, we tested whether the probability of ST sharing was influenced by the animals being sampled in the same month or by the same method, but none of these terms was significant in any of the models. While there might be temporal turnover of strains within hosts, the same strains could circulate within social groups, as has been shown for mountain brushtail possums [68].

It is possible that animals within a group only shared the same strains because they live in the same habitat. However, transmission from water sources on the ground can be excluded because sifakas do not drink from water holes. This is in contrast to a wetland elephant population, where infection from this environmental reservoir masked social factors [25]. Unfortunately, there is no empirical information on how long *E. coli* bacteria are able to survive on leaves and substrates used by sifakas, such as tree bark. However, light exposure in the dry deciduous Kirindy Forest is high, especially during the long dry season from April to October, and is accompanied by high-amplitude temperature fluctuations and little rainfall [41, 43], conditions which are regarded hostile for *E. coli* survival [42]. Conditions may be more favorable during the short wet season, however, which is characterized by more stable temperatures and regular rainfall.

Regarding transmission between different social groups, we found no significant influences on sharing the same ST. However, if sharing of single locus variants was included, both intergroup encounter rates and space-use sharing were significantly correlated with the transmission network. Thus, the effect of social structure on inter-group transmission became only apparent when we included evidence for less recent transmission events, indicating that the frequency of transmission between groups may be considerably lower than within groups. Unfortunately, we were not able to test the influence of specific social behaviors on transmission, due to disappearance of animals during the study period and a correspondingly low sample size of behavioral observations.

Because space sharing is a prerequisite for encounters to occur, encounter rate was expected and found to strongly correlate with our measure of range overlap, which takes into account the intensity of space use in the overlap area (UDOI). Consequently, we evaluated these parameters in two different Bayesian GLMMS. Both models had very similar deviance information criterion (DIC) values, indicating that neither model should be preferred over the other. Furthermore, the standardized effect sizes of intergroup encounter rates and UDOI regarding *E. coli* transmission were almost identical, making it difficult to determine whether transmission takes place predominantly through direct contact or through the subsequent use of the same substrates. We can thus not rule out environmental transmission. In contrast, association of individuals correlated with *E. coli* type sharing in giraffes, whereas home range overlap did not [26], but the intensity of use of the overlap area was not controlled for.

Most observed sifaka intergroup encounters did not involve physical contact. We recorded members of different groups to be in a distance of <1 m in only 33 % of observed encounters, and body contact occurred in only 16 % of them. Alternatively, we propose that transmission might be mediated by scent-marking. Sifakas scent-mark by rubbing their anogenital region and, in the case of males, chest glands on trees, and subsequent olfactory inspection of scent-marks and overmarking, i.e. placing a scent-mark directly on the scent-mark of another individual, are frequent behaviors in both male and female sifakas [69] and other lemurs [70]. Scent-marking has been shown to occur at higher rates in zones of home range overlap than in core areas [40] and occurred during 75.5 % of observed encounters. Recently, it has been shown that meerkats belonging to the same social group harbor similar bacterial communities in their anal scent glands, indicating that sociality plays a role in the acquisition of these bacterial assemblages [71]. Moreover, olfactory inspection of conspecific cues has been suggested as an explanation of *Salmonella* transmission in sleepy lizards [72]. Thus, olfactory communication involving signal inspection and overmarking, which are also common in other mammals [73, 74], and potentially other types of indirect species-specific social contacts are worth considering as mechanisms of social pathogen transmission in future studies of many other mammals.

Spillover of antimicrobial-resistant bacteria from humans into wildlife populations, which do not themselves come into contact with antimicrobial agents, has become an increasing concern, as these wild animal populations might constitute reservoirs for human infections [51, 75]. ESBL-producing *E. coli* have been found in wildlife populations ranging from seagulls and birds of prey to rodents and wild boar [reviewed in 51]. More recently, multidrug-resistant *E. coli* have been identified at high prevalence in banded mongoose living close to human settlements [45] and in a non-habituated gorilla living in a protected area [76].

Most *E. coli* isolates recovered in this study have not been described before in humans or domestic animals and were non-ESBL-producing. Thus, while we certainly did not sample the entire *E. coli* diversity harbored by sifakas, the data suggest that spillover of *E. coli* from humans into this population might be low, even though the study area is frequented regularly by tourists, researchers and occasionally villagers and livestock. We did not investigate *E. coli* carriage in humans in this study, but a survey including human stool samples from all over Madagascar revealed that more than 80 % of *E. coli* isolates were resistant to the most widely used antibiotics, including those tested in our study [57]. Bublitz et al. [44] recently found a higher prevalence of pathogenic *Enterobacteria* in lemurs living in habitats with frequent exposure to humans than in undisturbed forests. Lemurs found to be positive did not include *Propithecus sp*., however.

Nonetheless, we did recover ST131 isolates in 3 individuals. The spread of a single clonal group with an ST131 profile has largely been responsible for the sudden increase in ESBL-producing *E. coli* during the last decades and today constitutes the predominant lineage among extraintestinal pathogenic *E. coli* worldwide [77, 78]. Varying prevalences in healthy human populations suggest that ST131 may be a dominant strain also in healthy individuals [78]. ESBL-producing bacteria, including *E. coli* ST131, occur with high prevalence in the region of Antananarivo, the capital of Madagascar [79, 80]. The ST131 isolates which we recovered in this study were non-ESBL-producing, however. Additional susceptibility testing on all isolates belonging to this ST revealed full susceptibility to all antimicrobial agents tested. Along with some studies on humans, our results confirm that non-ESBL-producing, antibiotic-susceptible ST131 isolates exist [81, 82]. Another type known for ESBL-production, ST101, was also isolated, but was also non-ESBL-producing.

## Conclusions

In conclusion, our results provide suggestive evidence for socially-structured transmission of potentially pathogenic microorganisms within and between wild groups of primates. We showed that *E. coli* strain-sharing is most prevalent within groups of wild lemurs, while intergroup relationships affect population-wide spread. We propose that species-specific patterns of scent-marking and overmarking might constitute a likely route of social transmission, especially between groups. Further studies are needed to explicitly test this hypothesis and to further elucidate the relative roles of direct contact and environmental transmission. The absence of antibiotic resistance indicates that *E. coli* spillover into the population might be low, despite relatively high human impact, although spillover of other pathogens cannot be excluded. More detailed genetic analyses will be needed to clarify the extent to which the ST131 isolates discovered in this study are related to disease-causing human ST131 isolates.

## Additional files

10.1186/s12898-016-0059-y Isolates and host characteristics. A table including all of the *E. coli* isolates recovered in this study, together with information on their hosts, sampling date, and MLST profile.
10.1186/s12898-016-0059-y Dyadic home range overlap and intergroup encounter rates. A table of home range overlap and intergroup encounter rates per group-dyad.

## Abbreviations

DIC: deviance information criterion
*E. coli*: *Escherichia coli*
ESBL: extended-spectrum beta-lactamase
EUCAST: European Committee on Antimicrobial Susceptibility Testing
GLMM: general linear mixed model
GPS: global positioning system
h: hours
ha: hectare
km: kilometer
MALDI-ToF MS: matrix-assisted laser desorption ionization time-of-flight mass-spectrometry
min: minutes
MLST: multi-locus sequence typing
ST: sequence type
UDOI: utilization distribution overlap index
